# Subpopulation Particle Swarm Optimization with a Hybrid Mutation Strategy

**DOI:** 10.1155/2022/9599417

**Published:** 2022-02-23

**Authors:** Zixuan Xie, Xueyu Huang, Wenwen Liu

**Affiliations:** JiangXi University of Science and Technology, Ganzhou 341000, China

## Abstract

With the large-scale optimization problems in the real world becoming more and more complex, they also require different optimization algorithms to keep pace with the times. Particle swarm optimization algorithm is a good tool that has been proved to deal with various optimization problems. Conventional particle swarm optimization algorithms learn from two particles, namely, the best position of the current particle and the best position of all particles. This particle swarm optimization algorithm is simple to implement, simple, and easy to understand, but it has a fatal defect. It is hard to find the global optimal solution quickly and accurately. In order to deal with these defects of standard particle swarm optimization, this paper proposes a particle swarm optimization algorithm (SHMPSO) based on the hybrid strategy of seed swarm optimization (using codes available from https://gitee.com/mr-xie123234/code/tree/master/). In SHMPSO, a subpopulation coevolution particle swarm optimization algorithm is adopted. In SHMPSO, an elastic candidate-based strategy is used to find a candidate and realize information sharing and coevolution among populations. The mean dimension learning strategy can be used to make the population converge faster and improve the solution accuracy of SHMPSO. Twenty-one benchmark functions and six industries-recognized particle swarm optimization variants are used to verify the advantages of SHMPSO. The experimental results show that SHMPSO has good convergence speed and good robustness and can obtain high-precision solutions.

## 1. Introduction

So far, optimization algorithm has been a popular research problem. Particle swarm optimization algorithm is a population-based optimization algorithm, which was first invented by Dr. Eberhart and Dr. Kennedy in 1995 [1, 2]. Particle swarm optimization algorithm is inspired by the behavior of birds looking for food. Particle swarm optimization is a search algorithm in which particles cooperate with each other. Of course, particle swarm optimization algorithm is a population intelligent optimization algorithm. In addition to particle swarm optimization algorithm, common intelligent algorithms include differential evolution algorithm (DE) [3], ant colony optimization (ACO) algorithm [4], artificial bee colony (ABC) [5], programming algorithm (FEP) [6], simulated annealing algorithm [7], neural network [8], text clustering [9], resource allocation [10], and task allocation [11]. Particle swarm optimization algorithm has been widely accepted with the advantages of rapid convergence, excellent robustness, and concise understanding.

At present, many optimization algorithms have achieved significant results in many fields. It includes many applications, such as optimal tuning of type-1 and type-2 fuzzy controllers [12], optimal tuning of interval type-2 fuzzy controllers [13], scheduling planning [14], undergraduate systems engineering curriculum optimization technology [15], improving the performance of FinFET devices [16], brain models of fear processing and conflict modulation [17], multiobjective dynamic optimization problem [18, 19], mixed-variable Newsvendor problems [20], and feature selection [21].

With the advantages of particle swarm optimization algorithm [22], many applications use particle swarm optimization algorithm, such as image processing [23], neural network [24], feature selection [25], data clustering [26], and mixed-variable optimization problems (MVOPs) [27].

Although particle swarm optimization algorithm has been preeminently optimized and improved, it still has some problems to be solved. In particular, when the dimension of the population becomes higher, the particle swarm optimization algorithm is extremely prone to early convergence, which makes it hard to jump out of the local optimal solution in the final stage of the algorithm. In order to continue to improve the performance of particle swarm optimization algorithm, countless particle swarm optimization researchers are committed to four improvement directions, namely, particle swarm optimization parameter adjustment, learning strategy improvement, topology selection, and integration with other algorithms. These four aspects are described as follows:

Parameter control: it includes setting inertia weight, acceleration coefficient, and population size. The particle swarm optimization algorithm with constant inertia weight balances the ability of global development and local exploration. This method limits the particle swarm optimization algorithm in a sense. Another adaptive control parameter is proposed to control local balance and global exploration through adaptive parameters. Shi and Eberhart et al. proposed a PSO algorithm with an inertia weight set to a constant value to balance the global exploration and local exploitation abilities of the algorithm [28]. The inertia weight is constant, which greatly limits the potential search ability of particle swarm optimization. Zhan et al. proposed an APSO algorithm with adaptive control parameters. APSO achieves a locally and globally stable search state through adaptive control parameters [29].

Learning strategy improvement: it includes some comprehensive learning strategies, biogeography-based learning strategies, segmentation based advantage learning strategies, domain based learning strategies, and dynamic domain learning strategies. Yang et al. proposed the SPLSO algorithm that used segment-based predominant learning. This model first segments the dimensions of each poor-performing particle; then, each segment is learned from a better-performing particle, allowing the algorithm to capitalize on the information from the better particles and avoid premature convergence [30]. Liang and Suganthan proposed a DMS-PSO algorithm with a dynamic neighborhood structure in which the learning of each particle is no longer limited to one population; instead, it also includes other populations [31].

Topology: topology can effectively use particle information in the field, but it ignores the global optimal information to a certain extent. Common topologies include ring topology, star topology, network topology, dynamic tree topology, and dynamic competition topology. Li et al. proposed an adaptive particle swarm optimization algorithm using scale-free network topology. Based on the characteristics of scale-free network topology with a power-law distribution, the algorithm can construct a corresponding neighborhood for each particle [32]. Janson et al. constructed a dynamically changing tree topology in which each particle learns from its parent to utilize the information of each particle effectively [33].

Hybrid algorithm integration: mixing with other algorithms is one of the main research fields of particle swarm optimization algorithm, which can effectively improve the performance of the algorithm. Zhang et al. proposed the DSPSO algorithm by combining the differential mutation operation with the SLPSO algorithm [34]. Valdez et al. proposed a new hybrid approach for optimization by combining particle swarm optimization (PSO) and genetic algorithms (GAs) using fuzzy logic to integrate the results [35].

According to the above analysis, an excellent particle swarm optimization algorithm is to have better local exploration and global exploration capabilities. To achieve both, the diversity of population is essential, which can prevent the premature convergence of particle swarm optimization algorithm. Because each optimization problem is different, it is difficult for a single evolutionary strategy to meet each optimization problem. Relevant literature has shown that the combination of several strategies helps to improve the possibility of particle swarm convergence to global optimization. In order to adapt to more optimization problems, inspired by the MPCPSO algorithm, this paper proposed a joint strategy of subpopulation cooperative particle swarm optimization algorithm. The particle swarm is initialized, and the fitness values of all particles for the first time are recorded [36]. According to the fitness values, the population is divided into two populations: dominant population (DP) and poor population (PP). For the two populations, the first population and the second population adopt different evolutionary strategies, respectively, the second population adopts candidate learning strategy, and the first population adopts mean dimension learning. By comparing with other algorithms, the effect of the two strategies in this paper is feasible, and the solution of the function can be obtained at the same time.

The first section of this paper is organized as follows: Section 2 mainly introduces the related work of writing this article. Section 3 introduces the two learning strategies and SHMPSO in detail. In Section 4, the SHMPSO algorithm is tested by using twenty-one benchmark functions and six famous PSO variants. At the end of the article, Section 5 gives the relevant conclusions.

## 2. Related Work

This part mainly introduces some harvest before completing the experiment. Inspired by two particle swarm optimization algorithms, the first is the classical particle swarm optimization algorithm, and the second is the particle swarm optimization algorithm based on biogeography. Basically, all PSO variants are improved on the classic PSO.

### 2.1. Classic PSO

The classical particle swarm optimization algorithm is an optimization algorithm based on the whole population. Each particle represents the possibility of a solution. All particles update their positions according to their own historical best positions and global historical best positions. Generally, there are *D* dimensions in the search space of the whole particle swarm. The position vector of particle *i* at a certain time *t* is *X*_*i*_=(*x*_*i*1_, *x*_*i*2_,…*x*_*i*  *D*_), and the velocity vector is *V*_*i*_=(*v*_*i*1_, *v*_*i*2_,…*v*_*i*  *D*_). All particles of the next-generation population can be generated according to the position vector and velocity vector. The update equation for generating optimized next-generation particles is

(1) $$\begin{array}{c} V_{i\;d}=\omega V_{i\;d}\left(t\right)+c_{1}r_{1}\left(P_{i\;d}\left(t\right)-X_{i\;d}\left(t\right)\right)+c_{2}r_{2}\left(P_{g\;d}\left(t\right)-X_{i\;d}\left(t\right)\right), \end{array}$$

(2) $$\begin{array}{c} X_{i\;d}\left(t+1\right)=X_{i\;d}\left(t\right)+V_{i\;d}\left(t+1\right), \end{array}$$

where *ω* is an inertia weight, *c*_1_ and *c*_2_ are an acceleration factor, and *r*_1_ and *r*_2_ are a random number between [0, 1]. These two equations are the core equations of standard particle swarm optimization. The equation for speed and position is updated.

Although particle swarm optimization algorithm has made many improvements in recent decades, there is still an algorithmic barrier that is hard to breakthrough. For example, the algorithm is difficult to find the extreme value in the case of high dimension, and it is simple to fall into local optimization. At present, many strategies have been proposed to solve these difficulties. The following introduces the comprehensive learning strategies and biogeography learning strategies.

### 2.2. Particle Swarm Optimization Algorithm with the Comprehensive Learning Strategy

Liang et al. proposed a CLPSO algorithm, which is different from the conventional particle swarm optimization algorithm introduced earlier [37]. CLPSO never learns from the previously mentioned global optimal particle, but each dimension of the particle is like the historical optimal learning of the sample constructed in its field, and the learning probability is *P*_*c*_. CLPSO canonical learning scheme avoids falling into local optimization in some multimodal problems. The revised scheme proposed by CLPSO is described as follows:

(3) $$\begin{array}{c} V_{i\;d}\left(t+1\right)=\omega V_{i\;d}\left(t\right)+cr\left(P_{f_{i}\left(d\right)}\left(t\right)-X_{i\;d}\left(t\right)\right), \end{array}$$

where *ω* is the inertia weight, *c* is an acceleration coefficient, and *r* is a random number between (0, 1). *f*_*i*_(*d*) represents that in the *D* dimension, the particle *i* changes from *f*_*i*_(*d*) in *p* best that is an optimum position of particle history and *f*_*i*_(*d*)=[*f*_*i*_(1), *f*_*i*_(2),…, *f*_*i*_(*D*)]. All sample vectors of particle *i* are defined in *f*_*i*_(*d*). In CLPSO, equation (3) shows that each different particle can learn from different particles from different dimensions. The main core methods of CLPSO are shown in the following flow chart:

1. Generate random number *P* between (0, 1), and judge the size of *p* and *P*_*c*_.
2. If *P* > *P*_*c*_, the current particle *i* updates according to its optimal position.
3. If *P* < *P*_*c*_, the optimal particle is selected by comparing the fitness values of all particles. Let the particle replace the personal optimal position of the particle *i* and guide particle *i* to update.

The algorithm employs a comprehensive learning strategy whereby the best position before other particles is a paradigm that can be learned by any particle, and each dimension of a particle has the potential to learn from a different paradigm. The new strategy allows particles to have more learning paradigms and a larger potential flight space.

### 2.3. Learning Particle Swarm Optimization Algorithm Based on Biogeography (BLPSO)

BLPSO is improved according to CLPSO, which is based on biogeographic migration. BLPSO proposes a new learning strategy particle swarm optimization algorithm based on biogeography [38]. All particles in the population are updated by biogeographic migration using their own optimal location and the optimal location combination of other particles. All particles have a migration in and migration out rate, respectively, *α*_*i*_ and *β*_*i*_ to represent:

1. Sort each particle, and calculate the migration rate of each particle after sorting *α*_*i*_ and *β*_*i*_.
2. Generate the index of the sample vector,*f*_*i*_(*d*)=[*f*_*i*_(1), *f*_*i*_(2),…, *f*_*i*_(*D*)].
3. Generate a random number *r*. When *r* < *α*_*i*_, the index *j* sum of a particle and its migration rate is selected by the roulette selection probabilities *β*_*j*_. Assign *j* to *f*_*i*_. Otherwise, assign *i* to *f*_*i*_.
4. If *f*_*i*_ is equal to particle *i*, randomly select a particle *j* that is not equal to particle *i*, randomly select a dimension *l*, and assign the dimension of *j* to *f*_*i*_(*l*).

Contrastingly, the biogeography-based learning strategy employs a ranking technique whereby particles can learn more from particles with high-quality personal best positions, and this effectively enhances the exploitation of the original CLPSO.

## 3. Particle Swarm Optimization Algorithm with the Hybrid Strategy of Seed Swarm Optimization

The third section mainly introduces the particle swarm optimization algorithm based on the hybrid strategy of seed swarm optimization.  Section 3.1 mainly introduces the learning strategies of the mean dimension.  Section 3.2 mainly introduces the candidate generation strategy based on elasticity. The overall operation framework of this strategy will be given in Section 3.2. At the end of this section, the whole process of SHMPSO is shown in the form of pseudo code.

### 3.1. Learning Strategy of Mean Dimension

The particle swarm optimization algorithm for subpopulation has been around for a long time, and it has received excellent feedback on some issues. The CMPSODMO proposed by Liu et al. in 2017 handles multiobjective dynamic optimization problems in a complex and changing environment and uses a multiswarm-based particle swarm optimization framework to optimize problems in a dynamic environment [39]. The algorithm has achieved excellent results. In the FTPSO, proposed by Yazdani et al. in 2013, it was clearly proposed that in the algorithm, the advantages of multiple groups should be used to cover multiple peaks of the multiobjective problem in a dynamic space [40]. Inspired by TSLPSO, it was proposed by Xu et al. in 2019 [41]. TSLPSO proposes to adopt two populations and uses different strategies to iterate the two populations in the search space, respectively, and obtain significant results. Yen and Daneshyari proposed a method to exchange information among multiple swarms [42].

In the SHMPSO algorithm proposed in this paper, firstly, a population is initialized, and the population is divided into two populations by using the fitness ranking mechanism. The ranking order is ascending. In each iteration process of the algorithm, the part of particles with smaller fitness values is divided into the dominant population, and the other part is classified as the poor population. The proportional coefficient of the dominant population particle is *s*. The dominant populations use the mean dimension learning strategy to optimize the population, and the poor population uses the candidate generation strategy based on elasticity to learn. In this way, different evolutionary strategies for different populations can effectively ensure the diversity of populations and avoid falling into local optimization. The dominant population can guide the search direction of the whole population and make the population further converge to the solution quickly. There is also a strange phenomenon in PSO algorithm, which is called “spiral rise,” that is, the phenomenon of “two steps forward and one step backward” [43]. This means that although the fitness of the particles has been improved, the effects of the minority components of the particles have worsened. In order to overcome this problem, it is inevitable that the evaluation function needs to be changed frequently. Basically, all variants of PSO algorithm have some phenomena that the convergence speed is slow when the whole algorithm runs to the later stage. In SHMPSO, all particles are learning from the dominant population to achieve faster convergence speed.

Classical PSO requires two guiding particles, which are the current particle historical best value and the global historical best value. However, there are many algorithms that only have one guiding particle, such as the CLPSO and BLPSO mentioned above (Section 2). The advantage of this is that the speed update formula has fewer parameters and is easy to understand. The difficulty lies in how to construct this guide particle. For the dominant population, this article uses a guide particle. The following is an introduction to the evolution strategy of the dominant population.

For the whole dominant group, when the particle velocity is updated, the current particle will only receive the influence of *m* best generated by the comprehensive dimension learning.

The velocity update equation of particles is

(4) $$\begin{array}{c} {\text{V}}_{i\;d}\left(\text{t}+1\right)=\omega {\text{V}}_{i\;d}\left(\text{t}\right)+cr\left(m\;{\text{best}}_{i\;d}\left(t\right)-X_{i\;d}\left(t\right)\right), \end{array}$$

where *ω*, *c*, and *r* have the same meanings as in equation (3). Inspired by MPCPSO, *m* best_*i*  *d*_ is obtained from equation (5). Its purpose is to help particles get rid of the local optimal state. As can be seen from equation (9), *m* best_*i*  *d*_ is regarded as the only learning paradigm used to guide particle motion. Suppose the algorithm falls into the local extremum, *m* best_*i*  *d*_ seldom actively help particles find better solutions. In mean dimension learning, each particle learns not only from other particles but also from other related dimensions, which greatly increases the universality of particle domain learning.

(5) $$\begin{array}{c} m\;{\text{best}}_{i\;d}=\frac{\rho r}{D}\overset{D}{\underset{d=1}{\sum }}X_{i\;d}+\frac{\left(1-\rho \right)\left(1-r\right)}{N}\overset{N}{\underset{i=1}{\sum }}X_{i\;d}, \end{array}$$

where *D* represents the dimension of particles, *r* is a random number between [0, 1], and *N* represents the total number of particles in the dominant population. Among them, *ρ* is a dynamically defined value. The specific solution is as follows:

(6) $$\begin{array}{c} \rho =\frac{1}{1+\text{exp}\left(-\left(X_{i\;d}-1/D\sum_{d=1}^{D}X_{i\;d}\right)\right)}. \end{array}$$

The location update equation is as follows:

(7) $$\begin{array}{c} X_{i\;d}\left(t+1\right)=\phi X_{i\;d}\left(t\right)+V_{i\;d}\left(t+1\right), \end{array}$$

where *φ* is a dynamic parameter. Its equation (8) is given below:

(8) $$\begin{array}{c} \varphi =\frac{1}{{\left(1+\exp\left(-ave_{1}/N_{1}\sum_{i=1}^{N}f\left(x_{i}\right)\right)\right)}^{\text{iter}}}, \end{array}$$

where *N*_1_ is the current population size (the dominant population), *ave*_1_ refers to the average fitness of the dominant population after the first iteration of the population, and iter represents the current number of iterations. Based on the above iterative updating, the dominant population can be updated in a good direction.

It can be seen from equation (7) that the convergence speed of particles of the dominant population will be accelerated to a great extent. With the acceleration of convergence speed, it is inevitable that mean dimension learning is simple to fall into local optimization. In order to avoid this situation, a differential mutation operator is introduced to increase the diversity of the population [44, 45]. Here, we need an operation to randomly select two particles from the dominant population. The values of these two particles cannot be the same as those of the current iteration. At this time, we need to calculate the differential of these two random particles and make them a differential vector. This differential vector also needs to be mutated with the scaling factor of F. After mutation, it is summed with the global optimum (*P*_*g*  *d*_). The whole operation is described by the following equation:

(9) $$\begin{array}{c} X_{i\;d}\left(\text{t}+1\right)=P_{g\;d}\left(t\right)+F\left(X_{a}\left(t\right)-X_{b}\left(t\right)\right), \end{array}$$

where *P*_*g*  *d*_ is the global optimal position of the current population, *F* is a mutation coefficient. *a*  and *b* represent the index of randomly selected particles and meet the condition requirements *i* ≠ *a* ≠ *b*. The equation of mutation operation not only improves the search ability of the algorithm in the dominant particle swarm optimization but also expands the diversity in the process of population search.  Algorithm 1 lists the pseudo code of mean dimensions learning.

### 3.2. Candidate Generation Strategy Based on Elasticity

Next, the strategy of poor population is the candidate generation strategy based on elasticity. SHMPSO uses different populations and adopts different evolutionary strategies to evolve, respectively. This strategy is conducive to the rapid convergence of the population without losing the diversity of the population. The most important thing about this is how to generate candidates, which is the core part of the whole strategy. Here, the speed update equation has changed, instead of learning from the global optimization (*P*_*g*  *d*_) like the traditional particle swarm optimization algorithm, but introducing candidates and learning from candidates. New speed update equation is as follows:

(10) $$\begin{array}{c} V_{i\;d}\left(t+1\right)=\omega V_{i\;d}\left(t\right)+c_{1}r_{1}\left(p\text{ best }2_{i\;d}\left(t\right)-X_{i\;d}\left(t\right)\right)+c_{2}r_{2}\left({\text{Candidate}}_{i\;d}\left(t\right)-X_{i\;d}\left(t\right)\right), \end{array}$$

where *ω*, *c*_1_, *c*_2_, *r*_1_, and *r*_2_ have the same meanings as in equation (1). The vector *p* best 2_*i*  *d*_ represents the historical optimum of the current particle in the poor population. The generation method of Candidate_*i*  *d*_ is given below. Inspired by the elastic force generated by spring compression, an elastic coefficient prob is introduced here. The elastic coefficient of the poor population particle is set as prob=0.5. This parameter size is set by the user. Like a spring, it can be stretched or compressed. Particles are sorted in ascending order according to the fitness value, the larger the value, the worse the performance of the particles. A random number *r* between [0, 1] is randomly generated. If the elastic coefficient *prob* is greater than *r*, then the Candidate_*i*  *d*_ is generated by the equation:

(11) $$\begin{array}{c} {\text{Candidate}}_{id}=g\text{ best }1_{id}.*\text{step }{\text{zize}}_{id}*N, \end{array}$$

where the vector of *g* best 1_*id*_ represents the global optimal position of the dominant population. *N* represents the number of particles. The vector of step zize_*i*  *d*_ is a D-dimensional vector obtained by equation (17) and generated by Levy flight. An introduction to Levy flight is given below. The generation of Levy flight random number includes two parts. The first part is the selection of random direction, and the second part is the generation of Levy distribution. Random walks are derived from Levy stability. This distribution is a simple power-law equation:

(12) $$\begin{array}{c} L\left(s\right)∼{\left|s\right|}^{-1-\beta }, \end{array}$$

where 0 < *β* < 2. It is an index.

**Definition 1 .**

To determine Levy distribution mathematically, it defined by the following equation:

(13) $$\begin{array}{c} L\left(s,\gamma ,\mu \right)=\left\{\begin{array}{cc} \sqrt{\frac{\gamma }{2\pi }}\exp\left[-\frac{\gamma }{2\left(s-\mu \right)}\right]\frac{1}{{\left(s-\mu \right)}^{3/2}}, & \mathrm{if}\;0<\mu <s<\infty ,\\ 0, & \mathrm{if}\;s\leq 0, \end{array}\right) \end{array}$$

where the parameter *μ* is a control displacement parameter or position, *γ* > 0 represents the scale parameter (the scale used to control the distribution).

**Definition 2 .**

Usually, Levy distribution is defined by Fourier transform. The specific equation is given in the following:

(14) $$\begin{array}{c} \text{F}\left(\text{k}\right)=\exp\left[-\alpha {\left|\text{k}\right|}^{\beta }\right],0<\beta \leq 2, \end{array}$$

where *α* is a parameter in the interval [−1, 1], which is usually called skewness or scale factor. Stability index *β* is controlled between (0, 2), which is also commonly referred to as Levy index. *β* in most cases, his analytical form of integration is unknown. For random walking, the step *S* can be calculated by Mantegna's equation:

(15) $$\begin{array}{c} S=\frac{u}{{\left|v\right|}^{1/\beta }}, \end{array}$$

where *u* and *v* in equation (13) obey a positive distribution:

(16) $$\begin{array}{c} u∼N\left(0,\sigma_{u}^{2}\right),v∼N\left(0,\sigma_{u}^{2}\right),\\ \sigma_{u}={\left\{\frac{\tau \left(1+\beta \right)\sin\left(\pi \beta /2\right)}{\tau \left[1+\beta /2\right]\beta 2^{\left(\beta -1/2\right)}}\right\}}^{1/\beta }. \end{array}$$

The step size can then be calculated by the following equation:

(17) $$\begin{array}{c} \text{step size}=0.01\times S, \end{array}$$

where the factor 0.01 comes from the typical step factor *L*/100 , and *L* is a typical length ratio. Otherwise, Levy flight will become too radical and jump out of the design plan (waste evaluation).

When the elasticity factor prob < *r*, in order to ensure the diversity of the population, two particles *m*, *n* are selected from the dominant population. These two particles and the global optimal particle *P*_*g*  *d*_ are required which is different from each other. Compare particle *X*_*m*  *d*_ and *X*_*n*  *d*_ fitness value, and select the particle with a smaller fitness value *X*_*n*  *d*_. Candidate_*i*  *d*_ is obtained by the equation:

(18) $$\begin{array}{c} {\text{Candidate}}_{i\;d}=X_{n\;d}.*\text{step }{\text{zize}}_{i\;d}*N, \end{array}$$

where stepzize_*i*  *d*_ and N have the same meanings as in (12). The candidate improves the diversity of the population in this way.  Algorithm 2 lists the pseudo code of the candidate generation strategy based on elasticity.

### 3.3. Overall Framework of the SHMPSO Algorithm

Based on the improved strategies of 3.1 and 3.2, SHMPSO is constructed. The specific steps of the SHMPSO algorithm are as follows:

  Step 1: initialize the population and set the parameters, the mutation factor *F* = 0.5, the population proportion scale factor *s* = 0.5, and the times of falling into local optimum *M* = 6.
  Step 2: calculate the fitness value *f*(*X*_*i*_) and the global optimal value (*P*_*g*  *d*_) of all particles.
  Step 3: sorting in ascending order according to the fitness value, taking *s* ∗ *N* (all particles) particles as the dominant population (DP) and the rest particles as the poor population (PP)
  Step 4: optimizing disadvantaged populations based on flexible candidate strategy, which uses equations (2) and (10). The mean dimension learning is used to optimize the DP subpopulation through equations (4) and (7).
  Step 5: when *M* > 6, equation (9) is used to update the position of particles in the dominant population (DP).
  Step 6: recalculate the fitness values of all particles, and update the current global optimal value (*P*_*g*  *d*_).
  Step 7: repeat steps 3–6 when the maximum allowed times of iteration is bigger than the times of the maximum number of iterations.

**Figure 1:**
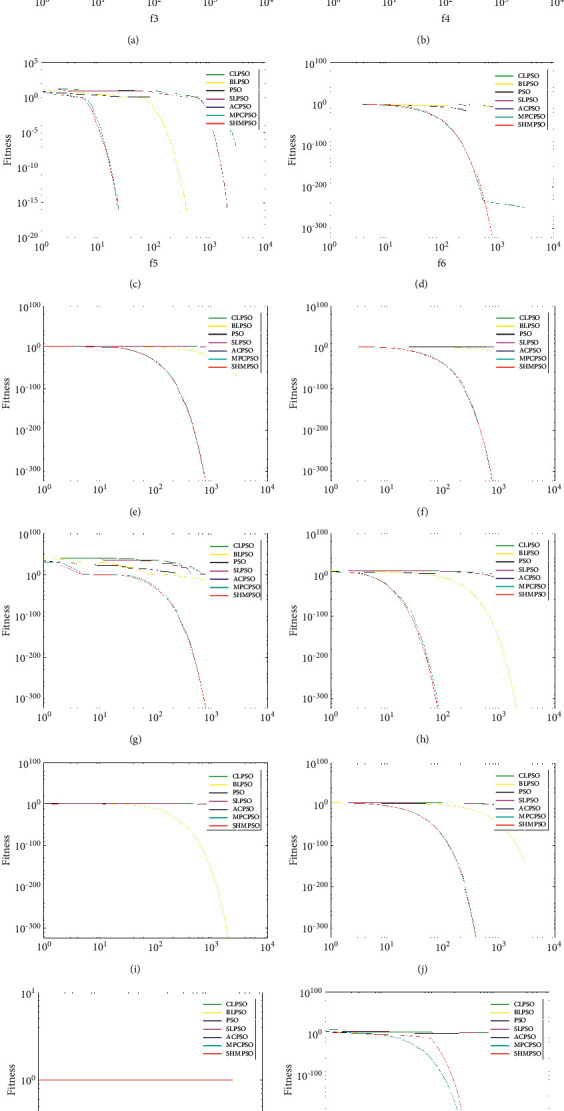
Convergence curves of 1–12 functions. (a) *f*_1_. (b) *f*_2_. (c) *f*_3_. (d) *f*_4_. (e) *f*_5_. (f) *f*_6_. (g) *f*_7_. (h) *f*_8_. (i) *f*_9_. (j) *f*_10_. (k) *f*_11_. (l) *f*_12_.

The algorithm flow chart of SHMPSO is shown in Figure 1. This algorithm mainly uses the global search ability to find a better search space. The algorithm starts from the global situation, finds the current global optimal position, and can converge to the global optimal solution faster. Thirdly, the general framework of the algorithm is given in Algorithm 3.

## 4. Experiment

In this section, in order to verify the reliability and efficiency of the proposed SHMPSO, twenty-one widely used benchmark functions are adopted. Comparing SHMPSO with other varieties of PSO, the results are verified. The experiment process is as follows.

### 4.1. Benchmark Function and Parameter Setting

The twenty-one benchmark functions listed in Table 1 are used to demonstrate the superiority of SHMPSO. In Table 1, the first column represents the function number, the second column represents the function name, the third column represents the function mathematical expression, the fourth column represents the function search range, and the fifth column shows the minimum value of the function. The tested functions include 11 unimodal functions (*f*_1_ − *f*_2,_*f*_4_ − *f*_12_) and 10 multimodal functions (*f*_3_, *f*_13_ − *f*_21_). The optimal value of all benchmark functions tested is 0. In order to compare the superior performance of the algorithm, six PSO variants are selected, including inertia weight PSO [40], ACPSO [46], SLPSO [47], CLPSO [37], BLPSO [38], and MPCPSO [36].

### 4.2. Selection of the Population Proportion Coefficient

In order to get a fairer comparison result, all the parameters used in the data experiment are the same, including the maximum number of independent runs (runNumber), the number of evaluations (maxFEs), and the maximum number of iterations (maxgen) where *N* represents the number of all particles in the population. All the comparison algorithms tested the twenty-one benchmark functions and got the mean and standard deviation after 30 runs. The parameter settings of all PSO variants are given in Table 2. According to the experimental results, all the functions are ranked and compared. In order to test the proportion of the dominant population in the SHMPSO algorithm, parameter *c* is tested, and the experimental results are as follows.

Through the overall analysis of Table 3 and ranking of each function, it is concluded that when *s* = 0.5, when the proportion of the dominant population in this paper is kept at 50%, the population optimization effect is the best. All the following comparisons are the experimental results based on the population scale coefficient of 0.5.

### 4.3. Comparison of Experimental Data between SHMPSO and Other PSO Variants

At the same time, 30, 50, and 100 dimensions are used to evaluate the given test function. The total population of SLPSO has its own definition, and all other functional population sizes are tested at (*N* = 100). When the particle dimension of all populations is 30 dimensions, maxgen is set to 3 × 10^3, and the function evaluation times maxFEs is set to 3 × 10^5. When the dimension of the particle is 50 dimensions, maxgen is set to 5 × 10^3, and the function evaluation times maxFEs is set to 5 × 10^5. When the particle dimension is 100 dimensions, the population's maxgen is set to 1 × 10^4, and the function evaluation times maxFEs is set to 1 × 10^6.

It can be seen from Tables 4 and 5 that the performance tested by the proposed SHMPSO is relatively stable in 30 and 50 dimensions, and the results obtained are similar. It can be seen that the performance of SHMPSO is excellent in the 30-dimensional and 50-dimensional convergence processes. SHMPSO is in *f*_1_, *f*_3_,  *f*_5_ − *f*_10_,  *f*_12_,  *f*_14_,  *f*_16_,  *f*_17_,  *f*_19_, and  *f*_20_. The optimal solution of these 14 functions can be found by the strategy proposed in this paper. These results explain that the strategy proposed in this paper can be well applied to these functions. Of course, the effects of *f*_15_ and *f*_21_ become worse with the increase of dimensions, and the optimization effect of SHMPSO on some multimodal functions which are difficult to optimize needs to be improved. In *f*_18_, the effect of SHMPSO is not as good as that of CLPSO, BLPSO, and SLPSO. However, in the overall 21 test functions, the average rank of SHMPSO ranks first, 1.61 and 1.67, respectively. Through the above analysis, the performance of SHMPSO is better than that of other six comparison algorithms.

In order to further verify the scalability and high efficiency of SHMPSO analysis, the proposed strategy is used to solve the 100-dimension problem, and the set parameters are the same as those in Table 4. The experimental results are shown in the following table.

In Table 6, it can be seen that SHMPSO still has high convergence accuracy and good robustness when solving high-dimensional problems. SHMPSO and MPCPSO rank first on the 100-dimensional problem. Further analysis, the performance of *f*_15_, *f*_18_, and *f*_21_ deteriorates drastically with increasing dimension. But the performance of other functions is still very good.

**Figure 2:**
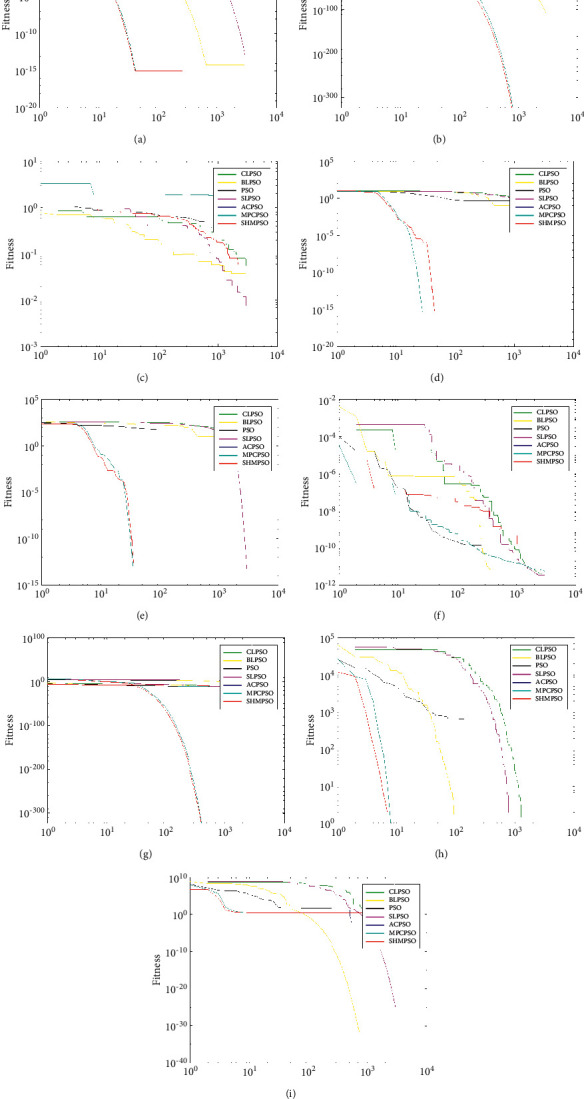
Convergence curves of 13–21 functions. (a) *f*_13_. (b) *f*_14_. (c) *f*_15_. (d) *f*_16_. (e) *f*_17_. (f) *f*_18_. (g) *f*_19_. (h) *f*_20_. (i) *f*_21_.

It can be seen from Figures 1 and 2 that the convergence performance of SHMPSO has obtained the global optimal value on most problems. Especially in *f*_3_, *f*_8,_*f*_13_, *f*_19_, *f*_20_, these five problems not only rank first in convergence accuracy but also the fastest convergence speed. Analyzing the reasons for convergence, there are three main reasons as follows. First of all, the information is highly shared among particles in the dominant population, and the guiding particles formed by the information sharing promote the dominant population to quickly converge to the global optimal value. Second, in order to prevent the population from falling into the local optimal value prematurely, a mutation operation is performed on the global optimal particle. Third, the use of random dominant population particles to guide the poor population particles not only improves the convergence speed of the poor population but also increases the diversity of the population. The particles of the dominant population are selected to guide the particles of the poor population, rather than the global optimal value of the poor population to guide the poor population. There are two reasons as follows: the first reason is that the fitness value of the poor population particles is larger than the fitness value of the population; the second reason is that the use of a single poor population global optimal particle is not conducive to increasing the diversity of the population.

According to the experiments in this paper, SHMPSO has good performance, and it has good performance in most functions. The success of SHMPSO mainly depends on the strategies proposed in this paper: First, based on the flexible candidate learning strategy, elite particles are selected from the dominant population to let the poor population particles learn, and the two populations share information, effectively jumping out of the local optimal solution. In addition, the mean dimension learning strategy can make the population particles have a better search range in complex and changeable multimodal functions, greatly improve the learning samples of particles, and provide more effective information for all particles. Therefore, SHMPSO has excellent properties and convergence accuracy.

## 5. Conclusion

Inspired by MPCPSO, this paper proposes a particle swarm optimization algorithm based on the strategy of subpopulation mixing. In this paper, the mean dimension learning strategy ensures the searching ability of the algorithm and the breadth of learnable samples, which provides the searching potential for the whole population. At the same time, the candidate learning strategy is used to improve the diversity of the population and prevent the population from falling into local optimum. At the same time, this paper compares SHMPSO with six well-known PSO variants to verify the effectiveness of SHMPSO proposed in this paper. Of course, SHMPSO still has some shortcomings, and *f*_2_ and *f*_11_ fall into local optimum. As the population searching ability needs to be improved, our future work will focus on the global searching ability of SHMPSO, and at the same time, we will deeply study the practical application of SHMPSO.

## Figures and Tables

**Algorithm 1:**
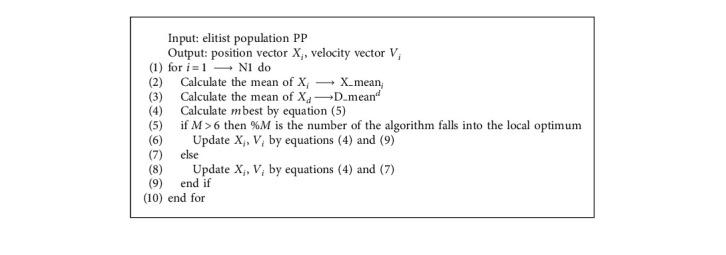
Mean learning strategy.

**Algorithm 2:**
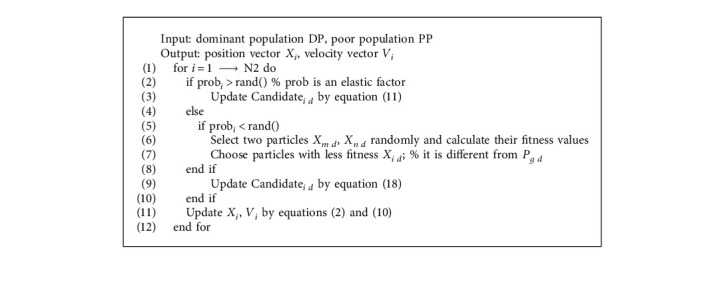
Strategy of candidate generation based on elasticity.

**Algorithm 3:**
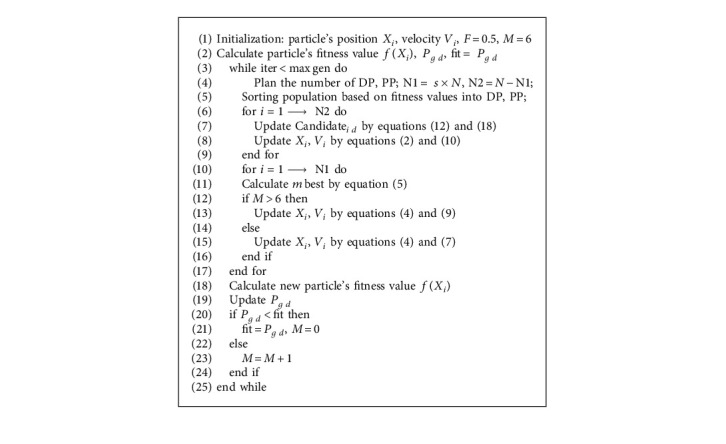
Grouping-mixed-based particle swarm optimization algorithm.

**Table 1:** Twenty-one benchmark functions.

Function	Function name	Test function	Search range	*f*_min_
*f*_1_	Brown function	∑_*i*=1_^*n*−1^(*x*_*i*_^2^)^(*x*_*i*+1_^2^+1)^+(*x*_*i*+1_^2^)^(*x*_*i*_^2^+1)^	[−1, 4]	0
*f*_2_	Exponential function	−exp(−0.5∑_*i*=1_^*n*^*x*_*i*_^2^)	[−1, 2]	0
*f*_3_	Griewank function	$1+\sum_{i=1}^{n}x_{i}^{2}/4000-\prod_{i=1}^{n}\text{cos}\left(x_{i}/\sqrt{i}\right)$	[−600, 600]	0
*f*_4_	Ridge function	*x*_*i*_+*d∗*(∑_*i*=1_^*n*^*x*_*i*_^2^)^*α*^, *d*=1, *α*=0.5	[−5, 5]	0
*f*_5_	Schwefel2.20 function	∑_*i*=1_^*n*^|*x*_*i*_|	[−100, 100]	0
*f*_6_	Schwefel2.21 function	$\underset{i=1,\ldots ,n}{\max}\left|x_{i}\right|$	[−100, 100]	0
*f*_7_	Schwefel2.22 function	∑_*i*=1_^*n*^|*x*_*i*_|+∏_*i*=1_^*n*^|*x*_*i*_|	[−100, 100]	0
*f*_8_	Schwefel2.23 function	∑_*i*=1_^*n*^*x*_*i*_^10^	[−10, 10]	0
*f*_9_	Sphere	∑_*i*=1_^*n*^*x*_*i*_^2^	[−5.12, 5.12]	0
*f*_10_	Sum squares	∑_*i*=1_^*n*^*ix*_*i*_^2^	[−10, 10]	0
*f*_11_	Xin-she Yang N.3	exp(−∑_*i*=1_^*n*^(*x*_*i*_/*β*)^2*m*^) − 2exp(−∑_*i*=1_^*n*^*x*_*i*_^2^)∏_*i*=1_^*n*^cos^2^(*x*_*i*_)	[−2 *π*, 2 *π*]	0
*f*_12_	Zakharou	∑_*i*=1_^*n*^*x*_*i*_^2^+(0.5*∗*∑_*i*=1_^*n*^*ix*_*i*_)^2^	[−5, 10]	0
*f*_13_	Ackley 1	$20+\exp\left(1\right)-20*\exp\left(0.2\sqrt{\sum_{i=1}^{n}x_{i}^{2}/n}\right)-\text{exp}\left[\frac{1}{n}\sum_{i=1}^{n}\cos\left(2\pi x_{i}\right)\right]$	[−35, 35]	0
*f*_14_	Alphine 1	∑_*i*=1_^*n*^|*x*_*i*_sin(*x*_*i*_)+0.1*x*_*i*_|	[0, 10]	0
*f*_15_	Happy cat	${\left(\sum_{i=1}^{n}x_{i}^{2}-\text{n}\right)}^{\alpha }+\frac{1}{n}\left(0.2\sum_{i=1}^{n}x_{i}^{2}+\sum_{i=1}^{n}x_{i}\right)+0.5$ , *α*=0.5	[−2, 2]	0
*f*_16_	Periodic	1+∑_*i*=1_^*n*^sin^2^(*x*_*i*_) − 0.1exp(−∑_*i*=1_^*n*^*x*_*i*_^2^)	[−10, 10]	0
*f*_17_	Rastrign	10n+∑_*i*=1_^*n*^[*x*_*i*_^2^ − 10cos(2*πx*_*i*_)]	[−5.12, 5.12]	0
*f*_18_	Xin-she Yang 2	(∑_*i*=1_^*n*^|*x*_*i*_|^*i*^)*∗*exp[−∑_*i*=1_^*n*^sin(*x*_*i*_^2^)]	[−2 *π*, 2 *π*]	0
*f*_19_	Schwefel 1.2	∑_*i*=1_^*n*^(*x*_1_+*x*_2_+⋯+*x*_*n*_)^2^	[−100, 100]	0
*f*_20_	Step2	∑_*i*=1_^*n*^*x*_*i*_+0.5^2^	[−100, 100]	0
*f*_21_	Penalized2	((sin^2^(3*πx*_*i*_)+∑_*i*=1_^*n*−1^(*x*_*i*_ − 1)^2^[1+sin^2^(3*πx*_*i*+1_)]+(*x*_*n*_ − 1)^2^)*∗*[1+sin^2^(2*πx*_*n*_)])*∗*0.1 + ∑_*i*=1_^*n*^100(*a*_*i*_ − 5)^4^, $a_{i}=\left\{\begin{array}{c} x_{i},x_{i}>5\\ -x_{i},x_{i}<-5 \end{array}\;\right)$	[−50, 50]	0

**Table 2:** Parameter settings of different PSO variants.

Algorithm	Parameter setting
PSO	*ω*=0.729, c1=c2=1.49445, *V*_max_=0.2 × range
ACPSO	*ω*=0.9 ~ 0.4, c1=c2=1.49445, *V*_max_=0.2 × range,alpha=0.1; beta=0.1
SLPSO	*m*=M+floor(*D*/100), c=*D*/*M∗*0.01, M=100
CLPSO	*ω*=0.9 ~ 0.2, gamp=5, c=1.49445, *V*_max_=0.2 × range
BLPSO	*ω*=0.9 ~ 0.2, gamp=5, I=*E*=1, *c*=1.49445, =0.2 × range
MPCPSO	*ω*=0.729, c1=c2=1.49445, *F*=0.5, *t*=0.5, *V*_max_=0.2 × range
SHMPSO	*ω*=0.729, c1=c2=1.49445, *F*=0.5, *s*=0.5, prob=0.5, *V*_max_=0.2 × range

**Table 3:** Selection of parameter *s*.

		*s* = 0.1	*s* = 0.2	*s* = 0.3	*s* = 0.4	*s* = 0.5	*s* = 0.6	*s* = 0.7
	Mean	0.00*E* + 00	0.00*E* + 00	0.00*E* + 00	0.00*E* + 00	0.00*E* + 00	0.00*E* + 00	0.00*E* + 00
*f*_1_	Std.	0.00*E* + 00	0.00*E* + 00	0.00*E* + 00	0.00*E* + 00	0.00*E* + 00	0.00*E* + 00	0.00*E* + 00
	Rank	1	1	1	1	1	1	1
	Mean	1.00*E* + 00	1.00*E* + 00	1.00*E* + 00	1.00*E* + 00	1.00 + 00	1.00*E* + 00	1.00*E* + 00
*f*_2_	Std.	6.78*E* – 16	6.78*E* − 16	6.78*E* – 16	6.78*E* − 16	6.78*E* − 16	6.78*E* − 16	6.78*E* − 16
	Rank	1	1	1	1	1	1	1
	Mean	0.00*E* + 00	0.00*E* + 00	0.00*E* + 00	0.00*E* + 00	0.00*E* + 00	0.00*E* + 00	0.00*E* + 00
*f*_3_	Std.	0.00*E* + 00	0.00*E* + 00	0.00*E* + 00	0.00*E* + 00	0.00*E* + 00	0.00*E* + 00	0.00*E* + 00
	Rank	1	1	1	1	1	1	1
	Mean	4.70*E* – 03	1.34*E* − 05	3.38*E* – 04	3.10*E* − 13	2.28*E* − 216	2.80*E* − 202	3.74*E* − 193
*f*_4_	Std.	2.57*E* – 02	5.10*E* − 05	1.80*E* – 03	1.70*E* − 12	0.00*E* + 00	0.00*E* + 00	0.00*E* + 00
	Rank	7	5	6	4	1	2	3
	Mean	0.00*E* + 00	0.00*E* + 00	0.00*E* + 00	0.00*E* + 00	0.00*E* + 00	0.00*E* + 00	0.00*E* + 00
*f*_5_	Std.	0.00*E* + 00	0.00*E* + 00	0.00*E* + 00	0.00*E* + 00	0.00*E* + 00	0.00*E* + 00	0.00*E* + 00
	Rank	1	1	1	1	1	1	1
	Mean	0.00*E* + 00	0.00*E* + 00	0.00*E* + 00	0.00*E* + 00	0.00*E* + 00	0.00*E* + 00	0.00*E* + 00
*f*_6_	Std.	0.00*E* + 00	0.00*E* + 00	0.00*E* + 00	0.00*E* + 00	0.00*E* + 00	0.00*E* + 00	0.00*E* + 00
	Rank	1	1	1	1	1	1	1
	Mean	0.00*E* + 00	0.00*E* + 00	0.00*E* + 00	0.00*E* + 00	0.00*E* + 00	0.00*E* + 00	0.00*E* + 00
*f*_7_	Std.	0.00*E* + 00	0.00*E* + 00	0.00*E* + 00	0.00*E* + 00	0.00*E* + 00	0.00*E* + 00	0.00*E* + 00
	Rank	1	1	1	1	1	1	1
	Mean	0.00*E* + 00	0.00*E* + 00	0.00*E* + 00	0.00*E* + 00	0.00*E* + 00	0.00*E* + 00	0.00*E* + 00
*f*_8_	Std.	0.00*E* + 00	0.00*E* + 00	0.00*E* + 00	0.00*E* + 00	0.00*E* + 00	0.00*E* + 00	0.00*E* + 00
	Rank	1	1	1	1	1	1	1
	Mean	0.00*E* + 00	0.00*E* + 00	0.00*E* + 00	0.00*E* + 00	0.00*E* + 00	0.00*E* + 00	0.00*E* + 00
*f*_9_	Std.	0.00*E* + 00	0.00*E* + 00	0.00*E* + 00	0.00*E* + 00	0.00*E* + 00	0.00*E* + 00	0.00*E* + 00
	Rank	1	1	1	1	1	1	1
	Mean	0.00*E* + 00	0.00*E* + 00	0.00*E* + 00	0.00*E* + 00	0.00*E* + 00	0.00*E* + 00	0.00*E* + 00
*f*_10_	Std.	0.00*E* + 00	0.00*E* + 00	0.00*E* + 00	0.00*E* + 00	0.00*E* + 00	0.00*E* + 00	0.00*E* + 00
	Rank	0	1	1	1	1	1	1
	Mean	1.00*E* + 00	1.00*E* + 00	1.00*E* + 00	1.00*E* + 00	1.00*E* + 00	1.00*E* + 00	1.00*E* + 00
*f*_11_	Std.	0.00*E* + 00	0.00*E* + 00	0.00*E* + 00	0.00*E* + 00	0.00*E* + 00	0.00*E* + 00	0.00*E* + 00
	Rank	1	1	1	1	1	1	1
	Mean	0.00*E* + 00	0.00*E* + 00	0.00*E* + 00	0.00*E* + 00	0.00*E* + 00	0.00*E* + 00	0.00*E* + 00
*f*_12_	Std.	0.00*E* + 00	0.00*E* + 00	0.00*E* + 00	0.00*E* + 00	0.00*E* + 00	0.00*E* + 00	0.00*E* + 00
	Rank	1	1	1	1	1	1	1
	Mean	8.88*E* – 16	8.88*E* − 16	8.88*E* – 16	8.88*E* − 16	8.88*E* − 16	8.88*E* − 16	8.88*E* − 16
*f*_13_	Std.	0.00*E* + 00	0.00*E* + 00	0.00*E* + 00	0.00*E* + 00	0.00*E* + 00	0.00*E* + 00	0.00*E* + 00
	Rank	1	1	1	1	1	1	1
	Mean	0.00*E* + 00	0.00*E* + 00	0.00*E* + 00	0.00*E* + 00	0.00*E* + 00	0.00*E* + 00	0.00*E* + 00
*f*_14_	Std.	0.00*E* + 00	0.00*E* + 00	0.00*E* + 00	0.00*E* + 00	0.00*E* + 00	0.00*E* + 00	0.00*E* + 00
	Rank	1	1	1	1	1	1	1
	Mean	1.07*E* + 00	4.47*E* − 01	4.51*E* – 01	5.90*E* − 01	2.19*E* − 01	1.01*E* + 00	1.75*E* − 01
*f*_15_	Std.	7.80*E* – 01	2.73*E* − 01	4.98*E* – 01	1.43*E* + 00	1.59*E* − 01	3.47*E* + 00	1.20*E* − 01
	Rank	7	3	4	5	2	6	1
	Mean	0.00*E* + 00	0.00*E* + 00	0.00*E* + 00	0.00*E* + 00	0.00*E* + 00	0.00*E* + 00	0.00*E* + 00
*f*_16_	Std.	0.00*E* + 00	0.00*E* + 00	0.00*E* + 00	0.00*E* + 00	0.00*E* + 00	0.00*E* + 00	0.00*E* + 00
	Rank	1	1	1	1	1	1	1
	Mean	0.00*E* + 00	0.00*E* + 00	0.00*E* + 00	0.00*E* + 00	0.00*E* + 00	0.00*E* + 00	0.00*E* + 00
*f*_17_	Std.	0.00*E* + 00	0.00*E* + 00	0.00*E* + 00	0.00*E* + 00	0.00*E* + 00	0.00*E* + 00	0.00*E* + 00
	Rank	1	1	1	1	1	1	1
	Mean	6.18*E* – 06	3.71*E* − 06	4.44*E* – 07	7.78*E* − 07	2.57*E* − 07	3.67*E* − 08	2.49*E* − 07
*f*_18_	Std.	7.85*E* – 06	7.21*E* − 06	1.54*E* – 06	3.19*E* − 06	6.04*E* − 07	1.10*E* − 07	1.34*E* − 06
	Rank	7	6	4	5	3	1	2
	Mean	0.00*E* + 00	0.00*E* + 00	0.00*E* + 00	0.00*E* + 00	0.00*E* + 00	0.00*E* + 00	0.00*E* + 00
*f*_19_	Std.	0.00*E* + 00	0.00*E* + 00	0.00*E* + 00	0.00*E* + 00	0.00*E* + 00	0.00*E* + 00	0.00*E* + 00
	Rank	1	1	1	1	1	1	1
	Mean	0.00*E* + 00	0.00*E* + 00	0.00*E* + 00	0.00*E* + 00	0.00*E* + 00	0.00*E* + 00	0.00*E* + 00
*f*_20_	Std.	0.00*E* + 00	0.00*E* + 00	0.00*E* + 00	0.00*E* + 00	0.00*E* + 00	0.00*E* + 00	0.00*E* + 00
	Rank	1	1	1	1	1	1	1
	Mean	2.62*E* + 00	2.89*E* + 00	3.10*E* + 00	2.81*E* + 00	2.85*E* + 00	2.83*E* + 00	2.96*E* + 00
*f*_21_	Std.	6.76*E* – 01	6.23*E* − 01	1.60*E* + 00	4.34*E* − 01	2.98*E* − 01	5.88*E* − 01	2.03*E* − 01
	Rank	1	5	7	2	4	3	6
Average rank		1.86	1.71	1.81	1.57	1.29	1.38	1.38

**Table 4:** Comparison of results of benchmark functions on various PSO variants (30-D).

		PSO	CLPSO	BLPSO	ACPSO	SLPSO	MPCPSO	SHMPSO
	Mean	1.34*E* + 01	9.66*E* − 11	5.10*E* − 30	1.00*E* – 11	2.08*E* − 140	0.00*E* + 00	0.00*E* + 00
*f*_1_	Std.	1.47*E* + 01	3.51*E* − 11	4.15*E* − 30	1.27*E* − 11	3.39*E* − 140	0.00*E* + 00	0.00*E* + 00
	Rank	7	6	4	5	3	1	1
	Mean	1.00*E* + 00	1.00*E* + 00	1.00*E* + 00	1.00*E* + 00	1.00*E* + 00	1.00*E* + 00	1.00*E* + 00
*f*_2_	Std.	6.78*E* − 16	9.38*E* − 13	4.65*E* − 16	0.00*E* + 00	6.78*E* − 16	6.78*E* − 16	6.78*E* − 16
	Rank	3	7	2	1	3	3	3
	Mean	1.05*E* + 00	1.89*E* − 07	0.00*E* + 00	5.48*E* − 02	5.75*E* − 04	0.00*E* + 00	0.00*E* + 00
*f*_3_	Std.	6.97*E* − 02	1.23*E* − 07	0.00*E* + 00	3.27*E* − 02	2.21*E* − 03	0.00*E* + 00	0.00*E* + 00
	Rank	7	4	1	6	5	1	1
	Mean	0.00*E* + 00	2.66*E* − 08	5.58*E* − 07	8.65*E* − 14	1.10*E* − 06	3.49*E* − 18	5.98*E* − 184
*f*_4_	Std.	0.00*E* + 00	4.12*E* − 08	8.24*E* − 07	1.22*E* − 13	1.72*E* − 06	1.91*E* − 17	0.00*E* + 00
	Rank	1	5	6	4	7	3	2
	Mean	3.13*E* + 01	8.20*E* − 05	1.24*E* − 16	1.50*E* − 04	2.70*E* − 70	0.00*E* + 00	0.00*E* + 00
*f*_5_	Std.	1.44*E* + 01	1.70*E* − 05	6.60*E* − 17	9.56*E* − 05	3.09*E* − 70	0.00*E* + 00	0.00*E* + 00
	Rank	7	5	4	6	3	1	1
	Mean	1.50*E* + 01	1.17*E* + 01	6.12*E* − 01	5.21*E* − 03	1.14*E* − 37	0.00*E* + 00	0.00*E* + 00
*f*_6_	Std.	3.55*E* + 00	9.31*E* − 01	2.62*E* − 01	4.08*E* − 03	1.09*E* − 37	0.00*E* + 00	0.00*E* + 00
	Rank	7	6	5	4	3	1	1
	Mean	2.10*E* + 02	1.17*E* − 04	1.49*E* − 16	1.70*E* − 04	3.17*E* − 69	0.00*E* + 00	0.00*E* + 00
*f*_7_	Std.	1.38*E* + 02	2.44*E* − 05	8.02*E* − 17	6.14*E* − 05	2.02*E* − 69	0.00*E* + 00	0.00*E* + 00
	Rank	7	5	4	6	3	1	1
	Mean	5.55*E* + 00	3.54*E* − 28	1.54*E* − 47	7.56*E* − 49	0.00*E* + 00	0.00*E* + 00	0.00*E* + 00
*f*_8_	Std.	1.17*E* + 01	6.44*E* − 28	5.49*E* − 47	2.67*E* − 48	0.00*E* + 00	0.00*E* + 00	0.00*E* + 00
	Rank	7	6	5	4	1	1	1
	Mean	0.00*E* + 00	2.62*E* − 11	2.75*E* − 23	1.50*E* − 11	0.00*E* + 00	0.00*E* + 00	0.00*E* + 00
*f*_9_	Std.	0.00*E* + 00	9.72*E* − 12	3.65*E* − 23	1.89*E* − 11	0.00*E* + 00	0.00*E* + 00	0.00*E* + 00
	Rank	1	7	5	6	1	1	1
	Mean	6.40*E* + 01	2.97*E* − 09	5.47*E* − 27	9.22*E* − 10	1.07*E* − 138	0.00*E* + 00	0.00*E* + 00
*f*_10_	Std.	3.67*E* + 01	1.08*E* − 09	5.16*E* − 27	9.09*E* − 10	1.81*E* − 138	0.00*E* + 00	0.00*E* + 00
	Rank	7	6	4	5	3	1	1
	Mean	1.00*E* + 00	1.00*E* + 00	1.00*E* + 00	1.00*E* + 00	1.00*E* + 00	1.00*E* + 00	1.00*E* + 00
*f*_11_	Std.	0.00*E* + 00	0.00*E* + 00	0.00*E* + 00	0.00*E* + 00	0.00*E* + 00	0.00*E* + 00	0.00*E* + 00
	Rank	1	1	1	1	1	1	1
	Mean	6.44*E* + 01	1.18*E* + 01	4.00*E* − 05	1.77*E* − 05	2.11*E* + 00	0.00*E* + 00	0.00*E* + 00
*f*_12_	Std.	3.60*E* + 01	2.35*E* + 00	4.30*E* − 05	2.46*E* − 05	2.27*E* + 00	0.00*E* + 00	0.00*E* + 00
	Rank	7	6	4	3	5	1	1
	Mean	8.53*E* + 00	1.48*E* − 04	2.95*E* − 14	1.88*E* − 05	5.74*E* − 15	8.88*E* − 16	8.88*E* − 16
*f*_13_	Std.	9.51*E* − 01	2.87*E* − 05	1.79*E* − 14	9.74*E* − 06	1.23*E* − 15	0.00*E* + 00	0.00*E* + 00
	Rank	7	6	4	5	3	1	1
	Mean	7.33*E* + 00	5.68*E* − 04	2.16*E* − 10	1.54*E* − 05	5.00*E* − 17	0.00*E* + 00	0.00*E* + 00
*f*_14_	Std.	2.56*E* + 00	1.13*E* − 04	5.15*E* − 10	9.12*E* − 06	1.31*E* − 16	0.00*E* + 00	0.00*E* + 00
	Rank	7	6	4	5	3	1	1
	Mean	2.89*E* − 01	5.33*E* − 02	6.84*E* − 03	8.89*E* − 03	4.42*E* − 02	7.36*E* + 00	2.19*E* − 01
*f*_15_	Std.	1.01*E* − 01	6.61*E* − 03	1.55*E* − 03	3.70*E* − 03	9.95*E* − 03	1.03*E* + 01	1.59*E* − 01
	Rank	6	4	1	2	3	7	5
	Mean	5.43*E* − 01	1.01*E* − 01	1.00*E* − 01	1.00*E* − 01	1.00*E* − 01	0.00*E* + 00	0.00*E* + 00
*f*_16_	Std.	2.53*E* − 01	3.44*E* − 04	2.94*E* − 06	3.21*E* − 06	1.13*E* − 16	0.00*E* + 00	0.00*E* + 00
	Rank	7	6	4	5	3	1	1
	Mean	6.91*E* + 01	1.14*E* − 04	0.00*E* + 00	2.48*E* − 07	1.34*E* + 01	0.00*E* + 00	0.00*E* + 00
*f*_17_	Std.	1.20*E* + 01	4.23*E* − 05	0.00*E* + 00	6.30*E* − 07	3.87*E* + 00	0.00*E* + 00	0.00*E* + 00
	Rank	7	5	1	4	6	1	1
	Mean	6.27*E* − 11	3.52*E* − 12	3.53*E* − 12	3.51*E* − 12	8.54*E* − 12	9.64*E* − 05	2.57*E* − 07
*f*_18_	Std.	4.87*E* − 11	1.33*E* − 15	8.58*E* − 14	5.05*E* − 19	1.45*E* − 12	2.81*E* − 04	6.04*E* − 07
	Rank	5	2	3	1	4	7	6
	Mean	1.68*E* + 03	2.38*E* + 03	1.33*E* + 01	7.40*E* − 02	1.72*E* − 11	0.00*E* + 00	0.00*E* + 00
*f*_19_	Std.	1.09*E* + 03	4.68*E* + 02	6.45*E* + 00	1.07*E* − 01	2.36*E* − 11	0.00*E* + 00	0.00*E* + 00
	Rank	6	7	5	4	3	1	1
	Mean	5.78*E* + 02	0.00*E* + 00	0.00*E* + 00	0.00*E* + 00	0.00*E* + 00	0.00*E* + 00	0.00*E* + 00
*f*_20_	Std.	2.18*E* + 02	0.00*E* + 00	0.00*E* + 00	0.00*E* + 00	0.00*E* + 00	0.00*E* + 00	0.00*E* + 00
	Rank	7	1	1	1	1	1	1
	Mean	8.23*E* + 01	3.95*E* − 08	2.71*E* − 26	3.95*E* + 07	1.35*E* − 32	2.91*E* + 00	2.82*E* + 00
*f*_21_	Std.	9.32*E* + 01	1.50*E* − 08	1.97*E* − 26	2.16*E* + 08	5.57*E* − 48	1.30*E* − 01	3.37*E* − 01
	Rank	6	3	2	7	1	5	4
Average rank		6	4.95	2.73	4.05	3.1	1.95	1.71

**Table 5:** Comparison of results of benchmark functions on various PSO variants (50-D).

		PSO	CLPSO	BLPSO	ACPSO	SLPSO	MPCPSO	SHMPSO
	Mean	4.38*E* + 01	8.38*E* − 11	8.18*E* − 35	3.50*E* − 12	8.09*E* − 160	0.00*E* + 00	0.00*E* + 00
*f*_1_	Std.	2.67*E* + 01	2.48*E* − 11	8.13*E* − 35	2.59*E* − 12	1.44*E* − 159	0.00*E* + 00	0.00*E* + 00
	Rank	7	6	4	5	3	1	1
	Mean	1.00*E* + 00	1.00*E* + 00	1.00*E* + 00	1.00*E* + 00	1.00*E* + 00	1.00*E* + 00	1.00*E* + 00
*f*_2_	Std.	2.26*E* − 16	6.21*E* − 16	3.66*E* − 16	0.00*E* + 00	4.46*E* − 16	2.26*E* − 16	2.26*E* − 16
	Rank	2	7	5	1	6	2	2
	Mean	1.58*E* + 00	2.19*E* − 08	0.00*E* + 00	2.43*E* − 02	1.64*E* − 03	0.00*E* + 00	0.00*E* + 00
*f*_3_	Std.	1.31*E* − 01	9.27*E* − 09	0.00*E* + 00	2.76*E* − 02	3.85*E* − 03	0.00*E* + 00	0.00*E* + 00
	Rank	7	4	1	6	5	1	1
	Mean	0.00*E* + 00	1.16*E* − 08	2.99*E* − 07	8.88*E* − 17	1.52*E* − 06	8.36*E* − 11	2.24*E* − 179
*f*_4_	Std.	0.00*E* + 00	1.78*E* − 08	2.24*E* − 07	2.71*E* − 16	1.11*E* − 06	4.58*E* − 10	0.00*E* + 00
	Rank	1	5	6	3	7	4	2
	Mean	7.91*E* + 01	9.12*E* − 05	1.04*E* − 19	1.34*E* − 04	9.39*E* − 81	0.00*E* + 00	0.00*E* + 00
*f*_5_	Std.	3.68*E* + 01	1.24*E* − 05	6.29*E* − 20	2.81*E* − 05	1.56*E* − 80	0.00*E* + 00	0.00*E* + 00
	Rank	7	5	4	6	3	1	1
	Mean	2.33*E* + 01	1.50*E* + 01	1.41*E* + 00	6.37*E* − 03	1.69*E* − 28	0.00*E* + 00	0.00*E* + 00
*f*_6_	Std.	2.60*E* + 00	1.10*E* + 00	2.77*E* − 01	2.11*E* − 03	3.90*E* − 28	0.00*E* + 00	0.00*E* + 00
	Rank	7	6	5	4	3	1	1
	Mean	9.10*E* + 02	1.38*E* − 04	5.29*E* − 20	1.50*E* − 04	2.00*E* − 79	0.00*E* + 00	0.00*E* + 00
*f*_7_	Std.	1.89*E* + 02	2.09*E* − 05	2.52*E* − 20	3.13*E* − 05	2.02*E* − 79	0.00*E* + 00	0.00*E* + 00
	Rank	7	5	4	6	3	1	1
	Mean	1.20*E* + 03	7.04*E* − 29	1.15*E* − 45	6.36*E* − 54	0.00*E* + 00	0.00*E* + 00	0.00*E* + 00
*f*_8_	Std.	1.44*E* + 03	7.10*E* − 29	4.92*E* − 45	2.80*E* − 53	0.00*E* + 00	0.00*E* + 00	0.00*E* + 00
	Rank	7	6	5	4	1	1	1
	Mean	4.00*E* + 01	8.82*E* − 11	3.24*E* − 23	6.60*E* − 12	0.00*E* + 00	0.00*E* + 00	0.00*E* + 00
*f*_9_	Std.	5.63*E* + 01	2.56*E* − 11	2.83*E* − 23	3.31*E* − 12	0.00*E* + 00	0.00*E* + 00	0.00*E* + 00
	Rank	7	6	4	5	1	1	1
	Mean	4.92*E* + 02	5.20*E* − 09	2.40*E* − 31	4.80*E* − 10	8.33*E* − 158	0.00*E* + 00	0.00*E* + 00
*f*_10_	Std.	1.75*E* + 02	1.15*E* − 09	3.14*E* − 31	2.07*E* − 10	2.17*E* − 157	0.00*E* + 00	0.00*E* + 00
	Rank	7	6	4	5	3	1	1
	Mean	1.00*E* + 00	1.00*E* + 00	1.00*E* + 00	1.00*E* + 00	1.00*E* + 00	1.00*E* + 00	1.00*E* + 00
*f*_11_	Std.	0.00*E* + 00	0.00*E* + 00	0.00*E* + 00	0.00*E* + 00	0.00*E* + 00	0.00*E* + 00	0.00*E* + 00
	Rank	1	1	1	1	1	1	1
	Mean	2.70*E* + 02	5.06*E* + 01	7.70*E* − 03	6.54*E* − 09	1.83*E* + 02	0.00*E* + 00	0.00*E* + 00
*f*_12_	Std.	9.69*E* + 01	7.82*E* + 00	4.95*E* − 03	3.77*E* − 09	3.04*E* + 01	0.00*E* + 00	0.00*E* + 00
	Rank	7	5	4	3	6	1	1
	Mean	1.18*E* + 01	1.06*E* − 04	6.45*E* − 15	1.01*E* − 05	6.34*E* − 15	8.88*E* − 16	8.88*E* − 16
*f*_13_	Std.	8.40*E* − 01	1.31*E* − 05	9.01*E* − 16	2.35*E* − 06	6.49*E* − 16	0.00*E* + 00	0.00*E* + 00
	Rank	7	6	4	5	3	1	1
	Mean	2.17*E* + 01	1.13*E* − 03	9.04*E* − 11	3.83*E* − 05	1.85*E* − 16	0.00*E* + 00	0.00*E* + 00
*f*_14_	Std.	5.10*E* + 00	1.74*E* − 04	4.94*E* − 10	1.70*E* − 05	2.62*E* − 16	0.00*E* + 00	0.00*E* + 00
	Rank	6	5	4	7	3	1	1
	Mean	5.13*E* − 01	7.88*E* − 02	1.24*E* − 02	1.01*E* − 02	1.29*E* − 01	6.22*E* + 00	2.96*E* + 00
*f*_15_	Std.	1.22*E* − 01	7.61*E* − 03	2.09*E* − 03	3.16*E* − 03	2.24*E* − 02	1.41*E* + 01	6.63*E* + 00
	Rank	5	3	2	1	4	7	6
	Mean	2.73*E* + 00	1.02*E* − 01	1.00*E* − 01	1.00*E* − 01	1.00*E* − 01	0.00*E* + 00	0.00*E* + 00
*f*_16_	Std.	8.90*E* − 01	4.24*E* − 04	2.40*E* − 06	6.10*E* − 05	7.06*E* − 17	0.00*E* + 00	0.00*E* + 00
	Rank	7	6	4	5	3	1	1
	Mean	1.87*E* + 02	2.30*E* − 04	3.79*E* − 15	1.87*E* − 07	3.01*E* + 01	0.00*E* + 00	0.00*E* + 00
*f*_17_	Std.	3.36*E* + 01	7.25*E* − 05	2.08*E* − 14	1.08*E* − 07	8.15*E* + 00	0.00*E* + 00	0.00*E* + 00
	Rank	7	5	3	4	6	1	1
	Mean	1.17*E* − 16	1.21*E* − 20	1.27*E* − 20	1.21*E* − 20	3.40*E* − 20	1.98*E* − 08	7.73*E* − 12
*f*_18_	Std.	2.25*E* − 16	7.47*E* − 24	6.13*E* − 22	2.18*E* − 26	4.04*E* − 21	7.77*E* − 08	3.83*E* − 11
	Rank	5	2	3	1	4	7	6
	Mean	8.29*E* + 03	1.21*E* + 04	3.47*E* + 02	1.26*E* − 02	2.69*E* − 03	0.00*E* + 00	0.00*E* + 00
*f*_19_	Std.	3.39*E* + 03	1.99*E* + 03	8.55*E* + 01	7.02*E* − 03	5.59*E* − 03	0.00*E* + 00	0.00*E* + 00
	Rank	6	7	5	4	3	1	1
	Mean	3.38*E* + 03	0.00*E* + 00	0.00*E* + 00	0.00*E* + 00	3.33*E* − 02	0.00*E* + 00	0.00*E* + 00
*f*_20_	Std.	9.39*E* + 02	0.00*E* + 00	0.00*E* + 00	0.00*E* + 00	1.83*E* − 01	0.00*E* + 00	0.00*E* + 00
	Rank	7	1	1	1	6	1	1
	Mean	5.43*E* + 04	2.43*E* − 08	1.20*E* − 30	6.50*E* − 06	1.46*E* − 03	4.88*E* + 00	4.89*E* + 00
*f*_21_	Std.	1.22*E* + 05	5.25*E* − 09	7.11*E* − 31	3.55*E* − 05	3.80*E* − 03	1.61*E* − 01	5.37*E* − 01
	Rank	7	2	1	3	4	5	6
Average rank		5.9	4.71	3.52	4.29	3.71	1.95	1.81

**Table 6:** Comparison of results of benchmark functions on various PSO variants (100-D).

		PSO	CLPSO	BLPSO	ACPSO	SLPSO	MPCPSO	SHMPSO
	Mean	4.10*E* + 02	2.01*E* − 11	9.65*E* − 40	2.73*E* − 07	8.53*E* − 179	0.00*E* + 00	0.00*E* + 00
*f*_1_	Std.	2.77*E* + 02	3.26*E* − 12	1.85*E* − 39	7.60*E* − 08	0.00*E* + 00	0.00*E* + 00	0.00*E* + 00
	Rank	7	5	4	6	3	1	1
	Mean	1.00*E* + 00	1.00*E* + 00	1.00*E* + 00	1.00*E* + 00	1.00*E* + 00	1.00*E* + 00	1.00*E* + 00
*f*_2_	Std.	0.00*E* + 00	0.00*E* + 00	0.00*E* + 00	0.00*E* + 00	0.00*E* + 00	0.00*E* + 00	0.00*E* + 00
	Rank	1	1	1	1	1	1	1
	Mean	3.82*E* + 00	1.15*E* − 09	1.11*E* − 17	9.76*E* − 03	9.86*E* − 04	0.00*E* + 00	0.00*E* + 00
*f*_3_	Std.	4.56*E* − 01	4.82*E* − 10	3.39*E* − 17	1.05*E* − 02	3.08*E* − 03	0.00*E* + 00	0.00*E* + 00
	Rank	7	4	3	6	5	1	1
	Mean	7.49*E* − 01	1.49*E* − 09	1.60*E* − 07	0.00*E* + 00	4.33*E* − 07	1.51*E* − 182	2.26*E* − 217
*f*_4_	Std.	5.53*E* − 01	1.88*E* − 09	2.31*E* − 07	0.00*E* + 00	3.96*E* − 07	0.00*E* + 00	0.00*E* + 00
	Rank	7	4	5	1	6	3	2
	Mean	3.31*E* + 02	5.58*E* − 05	1.94*E* − 24	2.24*E* − 02	2.67*E* − 91	0.00*E* + 00	0.00*E* + 00
*f*_5_	Std.	2.00*E* + 02	5.49*E* − 06	3.15*E* − 24	2.89*E* − 03	4.86*E* − 91	0.00*E* + 00	0.00*E* + 00
	Rank	7	5	4	6	3	1	1
	Mean	2.96*E* + 01	1.92*E* + 01	4.20*E* + 00	2.50*E* − 01	1.43*E* − 12	0.00*E* + 00	0.00*E* + 00
*f*_6_	Std.	2.80*E* + 00	6.32*E* − 01	5.58*E* − 01	3.29*E* − 02	1.02*E* − 12	0.00*E* + 00	0.00*E* + 00
	Rank	7	6	5	4	3	1	1
	Mean	1.95*E* + 03	9.77*E* − 05	5.23*E* − 25	2.21*E* − 02	1.23*E* − 89	0.00*E* + 00	0.00*E* + 00
*f*_7_	Std.	2.03*E* + 02	1.21*E* − 05	4.15*E* − 25	3.14*E* − 03	1.18*E* − 89	0.00*E* + 00	0.00*E* + 00
	Rank	7	5	4	6	3	1	1
	Mean	1.08*E* + 05	1.73*E* − 30	8.17*E* − 41	7.07*E* − 28	1.51*E* − 317	0.00*E* + 00	0.00*E* + 00
*f*_8_	Std.	9.88*E* + 04	1.04*E* − 30	1.90*E* − 40	6.30*E* − 28	0.00*E* + 00	0.00*E* + 00	0.00*E* + 00
	Rank	7	5	4	6	3	1	1
	Mean	2.30*E* + 02	1.64*E* − 10	2.77*E* − 17	5.44*E* − 07	0.00*E* + 00	0.00*E* + 00	0.00*E* + 00
*f*_9_	Std.	1.60*E* + 02	2.82*E* − 11	1.19*E* − 16	1.60*E* − 07	0.00*E* + 00	0.00*E* + 00	0.00*E* + 00
	Rank	7	5	4	6	1	1	1
	Mean	4.74*E* + 03	2.65*E* − 09	1.29*E* − 35	7.50*E* − 05	5.73*E* − 176	0.00*E* + 00	0.00*E* + 00
*f*_10_	Std.	1.36*E* + 03	5.23*E* − 10	4.69*E* − 35	2.55*E* − 05	0.00*E* + 00	0.00*E* + 00	0.00*E* + 00
	Rank	7	5	4	6	3	1	1
	Mean	1.00*E* + 00	1.00*E* + 00	1.00*E* + 00	1.00*E* + 00	1.00*E* + 00	1.00*E* + 00	1.00*E* + 00
*f*_11_	Std.	0.00*E* + 00	0.00*E* + 00	0.00*E* + 00	0.00*E* + 00	0.00*E* + 00	0.00*E* + 00	0.00*E* + 00
	Rank	1	1	1	1	1	1	1
	Mean	1.54*E* + 03	2.38*E* + 02	3.27*E* + 00	9.07*E* − 10	9.24*E* + 02	0.00*E* + 00	0.00*E* + 00
*f*_12_	Std.	4.11*E* + 02	1.98*E* + 01	8.08*E* − 01	4.46*E* − 10	9.08*E* + 01	0.00*E* + 00	0.00*E* + 00
	Rank	7	5	4	3	6	1	1
	Mean	1.44*E* + 01	3.30*E* − 05	1.46*E* − 14	1.70*E* − 03	1.38*E* − 14	8.88*E* − 16	8.88*E* − 16
*f*_13_	Std.	5.44*E* − 01	2.68*E* − 06	2.87*E* − 15	2.56*E* − 04	4.04*E* − 15	0.00*E* + 00	0.00*E* + 00
	Rank	7	5	4	6	3	1	1
	Mean	6.11*E* + 01	2.45*E* − 03	6.56*E* − 16	7.36*E* − 03	1.16*E* − 15	0.00*E* + 00	0.00*E* + 00
*f*_14_	Std.	7.72*E* + 00	2.57*E* − 04	1.47*E* − 15	1.32*E* − 03	1.02*E* − 15	0.00*E* + 00	0.00*E* + 00
	Rank	7	5	3	6	4	1	1
	Mean	6.90*E* − 01	1.49*E* − 01	2.59*E* − 02	2.29*E* − 02	2.79*E* − 01	1.88*E* + 00	3.27*E* + 00
*f*_15_	Std.	9.15*E* − 02	1.46*E* − 02	2.70*E* − 03	3.60*E* − 03	3.54*E* − 02	4.41*E* − 02	9.98*E* + 00
	Rank	5	3	2	1	4	6	7
	Mean	1.23*E* + 01	1.04*E* − 01	1.00*E* − 01	1.51*E* − 01	1.00*E* − 01	0.00*E* + 00	0.00*E* + 00
*f*_16_	Std.	2.63*E* + 00	6.29*E* − 04	4.18*E* − 06	1.13*E* − 02	1.07*E* − 16	0.00*E* + 00	0.00*E* + 00
	Rank	7	5	4	6	3	1	1
	Mean	5.54*E* + 02	3.63*E* − 04	2.98*E* − 01	1.14*E* − 01	1.08*E* + 02	0.00*E* + 00	0.00*E* + 00
*f*_17_	Std.	3.99*E* + 01	7.70*E* − 05	5.32*E* − 01	4.63*E* − 02	2.79*E* + 01	0.00*E* + 00	0.00*E* + 00
	Rank	7	3	5	4	6	1	1
	Mean	7.45*E* − 28	4.68*E* − 42	6.04*E* − 42	5.51*E* − 42	1.62*E* − 41	4.49*E* − 11	1.68*E* − 18
*f*_18_	Std.	3.03*E* − 27	4.16*E* − 45	3.89*E* − 43	5.39*E* − 43	1.49*E* − 42	2.39*E* − 10	9.22*E* − 18
	Rank	5	1	3	2	4	7	6
	Mean	3.71*E* + 04	6.84*E* + 04	1.30*E* + 04	9.40*E* + 04	1.61*E* + 04	0.00*E* + 00	0.00*E* + 00
*f*_19_	Std.	1.14*E* + 04	5.96*E* + 03	1.84*E* + 03	5.15*E* + 05	7.73*E* + 03	0.00*E* + 00	0.00*E* + 00
	Rank	5	6	3	7	4	1	1
	Mean	1.67*E* + 04	0.00*E* + 00	0.00*E* + 00	0.00*E* + 00	4.67*E* − 01	0.00*E* + 00	0.00*E* + 00
*f*_20_	Std.	2.68*E* + 03	0.00*E* + 00	0.00*E* + 00	0.00*E* + 00	1.66*E* + 00	0.00*E* + 00	0.00*E* + 00
	Rank	7	1	1	1	6	1	1
	Mean	2.49*E* + 06	6.38*E* − 09	2.15*E* − 30	1.67*E* + 08	2.56*E* − 03	9.82*E* + 00	9.95*E* + 00
*f*_21_	Std.	1.56*E* + 06	1.11*E* − 09	7.87*E* − 30	9.17*E* + 08	4.73*E* − 03	2.31*E* − 01	2.38*E* − 01
	Rank	6	2	1	7	3	4	5
Average rank		5.1	3.9	3.29	4.38	3.57	1.76	1.76

## Data Availability

All data of the paper can be obtained through the corresponding author upon request.
